# Surface topology affects wetting behavior of *Bacillus subtilis* biofilms

**DOI:** 10.1038/s41522-017-0018-1

**Published:** 2017-04-25

**Authors:** Moritz Werb, Carolina Falcón García, Nina C. Bach, Stefan Grumbein, Stephan A. Sieber, Madeleine Opitz, Oliver Lieleg

**Affiliations:** 1 Department of Mechanical Engineering and Munich School of Bioengineering, Technische Universität München, Garching, Germany; 2 Department of Chemistry, Chair of Organic Chemistry II, Center for Integrated Protein Science Munich (CIPSM), Technische Universität München, Garching, Germany; 3 Center for NanoScience, Faculty of Physics, Ludwig-Maximilians-Universität München, München, Germany

## Abstract

The colonization of surfaces by bacterial biofilms constitutes a huge problem in healthcare and industry. When attempting biofilm inactivation or removal, it is crucial to sufficiently wet the biofilm surface with antibacterial agents; however, certain biofilms efficiently resist wetting, and the origin of this behavior remains to date unclear. Here, we demonstrate that, depending on the growth medium used, the model bacterium *Bacillus subtilis* can form biofilm colonies with distinct surface properties: we find either hydrophilic or two variants of hydrophobic behavior. We show that those differences in biofilm wetting correlate with distinct surface topologies which, in turn, give rise to different physical wetting regimes known from lotus leaves or rose petals. Forming biofilms with different wetting properties may help bacteria to survive in both arid and humid conditions. Furthermore, converting the surface polarity of a biofilm could facilitate their removal from surfaces by increasing their wettability.

## Biofilm structure: Surface control of wetting

A biofilm’s surface structure affects its susceptibility to wetting, influencing persistence and suggesting strategies for biofilm removal. Effective wetting of biofilm surfaces is essential for combatting them with antibacterial agents. Resistance to the procedures currently available for removing biofilms is a huge problem in healthcare. Researchers in Germany led by Oliver Lieleg at the Technical University of Munich found that *Bacillus subtilis* bacteria can form colonies with different surface structures that either resist or assist wetting. This structural variability in their biofilms may allow the bacteria to adapt to survive in either arid or humid conditions. The insight that environmental conditions can influence biofilm wettability should spur research to control this natural variation. Learning how to convert biofilms into more readily wetted forms may greatly assist their removal in healthcare situations.

## Introduction

In nature, a broad range of biological materials have evolved to repel liquids. Lotus^1^ and rice leaves,^2^ rose petals,^3^ gecko’s feet,^4^ the legs of the water strider,^5^ and insect wings,^6^ have revealed well-orchestrated physical mechanisms that dictate their wetting resistance. Their extraordinary surface properties make them attractive for environmental,^7^ industrial,^8, 9^ technological,^10^ and biomedical,^11, 12^ applications.

Lotus-like superhydrophobic surfaces (SHS) possess contact angles towards water larger than 150°, low-contact angle hysteresis, and are characterized by the formation of a composite solid-liquid-air interface—a key mechanism that allows impacting^13, 14^ and condensed water droplets to bounce-off or roll-off easily^15^ (Cassie–Baxter wetting state^16^). Artificial superhydrophobic materials mimic surface structures found on biological templates:^17–19^ SHS inspired by the lotus leaf exhibit roughness features on both the nanoscale and microscale, and are often combined with low surface energy materials or coatings.^2, 20–22^

Another type of superhydrophobic behavior is found on rose petals. Here, contact angles with water are similarly high, but water droplets remain adhered to the petal surface when tilted.^3^ There are also surfaces which prevent ice adhesion^23^ (icephobic surfaces) and others can repel both polar and apolar liquids^24, 25^ (omniphobic surfaces).

An example of a biological surface which repels not only water but even water/solvent mixtures is given by bacterial biofilms. Biofilms are viscoelastic materials comprising bacteria and secreted macromolecules. By embedding themselves into a biopolymer matrix, the bacteria are protected from harsh environmental conditions. Biofilms formed by the model bacterium *Bacillus subtilis* resist liquid wetting up to 80% ethanol,^26^ a mechanism which severely limits its antibacterial efficiency. The amphiphilic protein Bacillus surface layer protein A (BslA) has been shown to contribute to the water repellency of *B. subtilis* biofilms by forming a hydrophobic surface layer and increasing the micro-roughness of the biofilm surface.^27^ For mutant strains unable to produce BslA, the biofilm colonies were observed to be hydrophilic. Although this remarkable wetting resistance of biofilms may be a key reason why bacteria are that resilient towards antimicrobials,^28^ biocides, and solvents, the underlying physical principles giving rise to this superhydrophobic behavior are still not fully understood. In particular, a direct correlation of physical wetting regimes as described by Wenzel^29^ and Cassie–Baxter^16^ with differences in the wetting behavior of biofilms has not been established yet. This is mainly due to a lack of suitable measuring methods that allow for quantitatively comparing the surfaces of soft biological materials such as biofilms.

A topological characterization of surfaces is commonly performed using scanning electron microscopy (SEM) imaging. However, for soft biological materials such as bacterial biofilms, the required sample preparation procedure may alter the material properties. Also, SEM images are mostly limited to providing qualitative information. A complementary technique for the topological characterization of biofilm surfaces is confocal fluorescence microscopy. With this technique, a 3D image of the material is obtained that provides information on biofilm thickness, surface area coverage, and surface roughness.^30, 31^ However, a more detailed analysis of the surface topology of bacterial biofilms is typically not performed. Recently, white light profilometry has been shown to possess great potential as a new non-destructive imaging technique for the visualization of bacterial biofilms in situ.^32^ Still, data obtained with this technique has so far mainly been analyzed in terms of sample thickness and roughness.^32–34^ In contrast, the surface topology of lotus leaves and rose petals has already been quantified in great detail, e.g., using both traditional and more complex metrological parameters.^35^

Here, we show that biofilms generated by the bacterium *B. subtilis* NCIB 3610 can exhibit three different modes of wetting.^36^ Depending on both the growth medium used for biofilm generation and the location on the biofilm colony, we find a hydrophilic and two hydrophobic biofilm variants, i.e., water repellent surfaces with either strong or weak water droplet adhesion. Using a combination of imaging techniques, we correlate those different wetting behaviors with structural differences of the biofilm surfaces, which we quantify with metrological parameters. Furthermore, we demonstrate that the two hydrophobic biofilm variants can be described by different physical wetting regimes that are related to the lotus and rose petal effect, and that the distinct wetting properties of the biofilms are accompanied by alterations in the biofilm matrix composition.

## Results and discussion

### Wetting behavior of *B. subtilis* NCIB 3610 biofilms grown on different agar variants

**Fig. 1:**
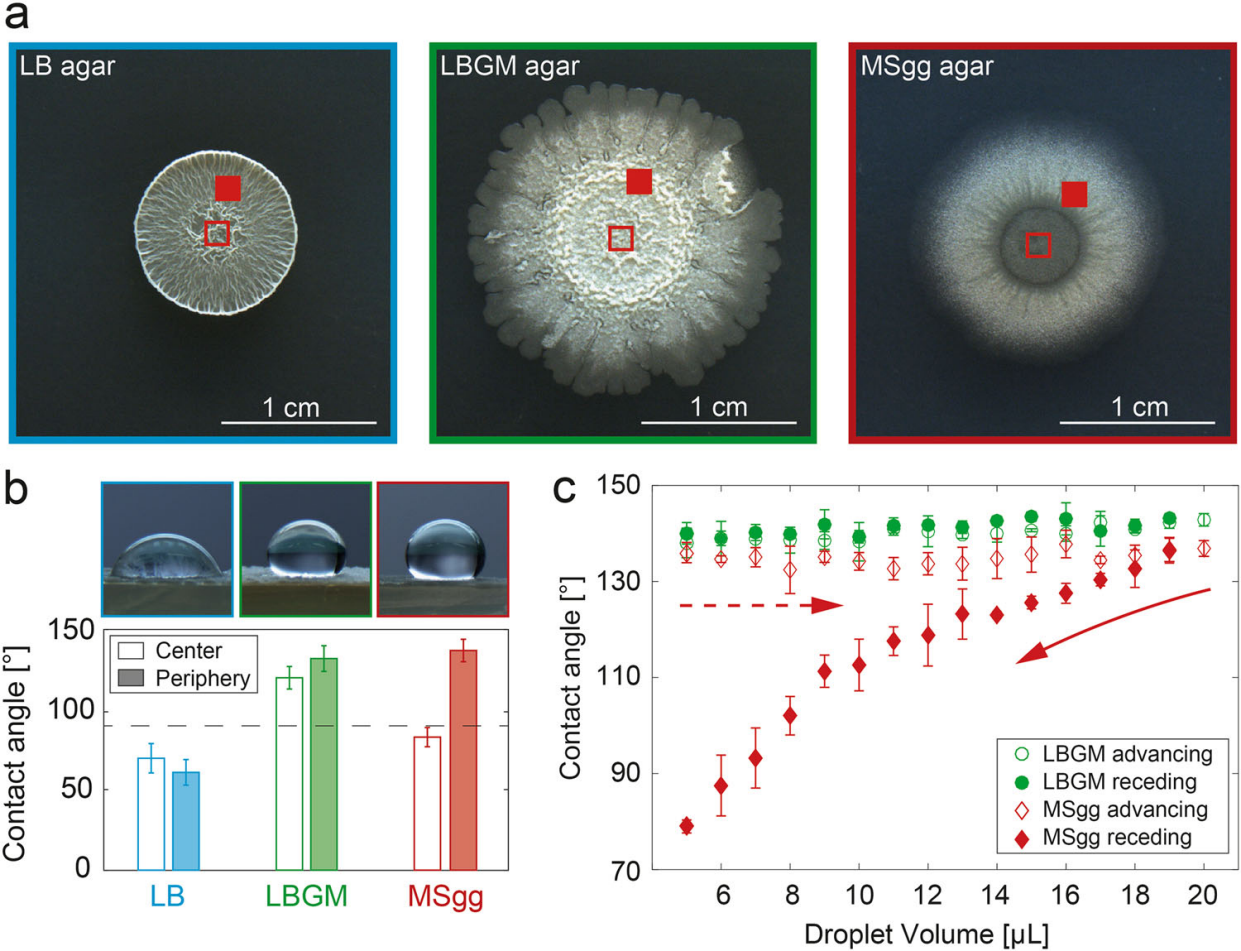
The wetting behavior of *B. subtilis* NCIB 3610 biofilms depends on the biofilm growth medium and on the location on the biofilm colony. When *B. subtilis* NCIB 3610 is grown on LB agar enriched with different molecules, the morphology of the formed macrocolonies changes **a** and a different wetting behavior of the biofilms is observed (**b**). In the images shown in **a**, the regions on the biofilm surface where the wetting tests were performed are marked with a *closed* and *open red square*, respectively. The droplet images shown in **b** were acquired on the peripheral regions of the biofilm colonies. For the peripheral regions of biofilms grown on MSgg agar, a pronounced contact angle hysteresis is observed, but not for biofilms grown on LBGM agar (**c**). The experimental time scale for the wetting/dewetting experiment was identical for both biofilm variants. Error bars denote the standard deviation. For data shown in **b**, *n* ≥ 9; for data shown in **c**, *n* = 3

When bacteria of the strain *B. subtilis* NCIB 3610 are cultivated on standard Luria Miller broth (LB) agar, the biofilm colonies formed exhibit a fairly homogenous morphology with delicate vein-like structures branching out from the center to the peripheral region of the colony. In contrast, the biofilm colonies grown on LBGM agar (i.e., LB agar enriched with 100 µM Manganese(II)sulfate (MnSO4) and 1% glycerol)^37^ show aerial projections enclosing the center region and appear Eden-like with dense branching at the edge of the colony. Biofilm grown on MSgg agar (i.e., minimal agar containing a complex combination of multivalent ions and amino acids, see Methods for details) shows overall a wrinkled morphology but with a smoother texture in the center (Fig. 1a). The biofilm colony morphologies found here differ slightly from those described in the literature^27, 38–41^ as the growth temperature and growth time used in our study are different.

For probing the wetting behavior of those three biofilm variants, a 10 µL water droplet is placed onto the biofilms, and the static contact angle is determined. For biofilm colonies grown on standard LB-agar, a contact angle of (61 ± 8)° is obtained, (Fig. 1b) which corresponds to hydrophilic behavior. Such a low wetting resistance is observed at virtually all locations of the biofilm, i.e., both in the center and the peripheral regions of the colony. In contrast, the peripheral regions of the other two biofilm variants both show hydrophobic behavior: we measure contact angles of (132 ± 8)° for the biofilm grown on LBGM agar and (137 ± 7)° for the biofilm grown on MSgg agar (Fig. 1b). Similarly high-contact angle values are also obtained for 50/50 mixtures of water and alcohols (Extended Data Table 1), which is consistent with previous findings for *B. subtilis* biofilms grown on MSgg agar.^26^ Although the peripheral regions of both the LBGM and the MSgg biofilm show hydrophobic properties, the wetting behavior of the central regions of those two biofilm variants differ: In the center of the MSgg grown biofilm, we find rather hydrophilic behavior (Extended Data Fig. 1) with a contact angle of only (83 ± 6)°. In some cases it appears that the water droplet slips below the central area of the MSgg biofilm and detaches the biofilm from the agar layer. In contrast, in the central region of the LBGM grown biofilm, we measure a contact angle of (120 ± 7)° which clearly indicates a hydrophobic surface (Fig. 1b).

A strong wetting resistance is observed on a broad range of natural as well as artificial materials and can be further classified into lotus leaf-like and rose petal-like behavior.^3, 42^ On a lotus leaf, very high-contact angles up to 150° are observed, and water droplets easily roll off the surface when the leaf is slightly tilted.^15^ In contrast, a hydrophobic surface which exhibits strong adhesion forces towards a water droplet is, for example, found on rose petals.^43^ Here, these strong adhesion forces prevent a small water droplet from rolling off the surface of the rose petal—even if the petal surface is tilted or turned upside down (Extended Data Fig. 2). At the same time, such rose petal surfaces show a hysteresis in the contact angle, i.e., a constant contact angle when the volume of a wetting water droplet is increased, but a decreasing contact angle when the volume of this droplet is reduced again. Thus, in a next step, we further characterize the wetting behavior of the two hydrophobic biofilm variants. We first place a small water droplet onto the biofilms and then gradually increase the volume of the water droplet from 5 µL to 20 µL (Fig. 1c). Afterwards, we step by step decrease the volume of the water droplet back to 5 µL. For the LBGM biofilm variant, the contact angle of the droplet remains virtually constant during this process. Furthermore, when we tilt the surface of the LBGM grown biofilm, the water droplet easily rolls off the biofilm surface. These results motivate that the wetting behavior of *B. subtilis* 3610 biofilms grown on LBGM is related to that of lotus-leaves, i.e., hydrophobic without any perceivable contact angle hysteresis. The very same bacteria, however, are able to form biofilms with rose petal-like wetting behavior when grown on MSgg agar: the MSgg biofilm sample can be tilted vertically and the water droplet stays attached to the surface (Extended Data Fig. 2). Also, we find a pronounced contact angle hysteresis for the MSgg biofilm: the contact angle remains constant when the volume of the water droplet is increased (advancing contact angle), but the contact angle continuously decreases when the water droplet volume is lowered again (receding contact angle).

### Quantification of surface topology of *B. subtilis* NCIB 3610 biofilms

**Fig. 2:**
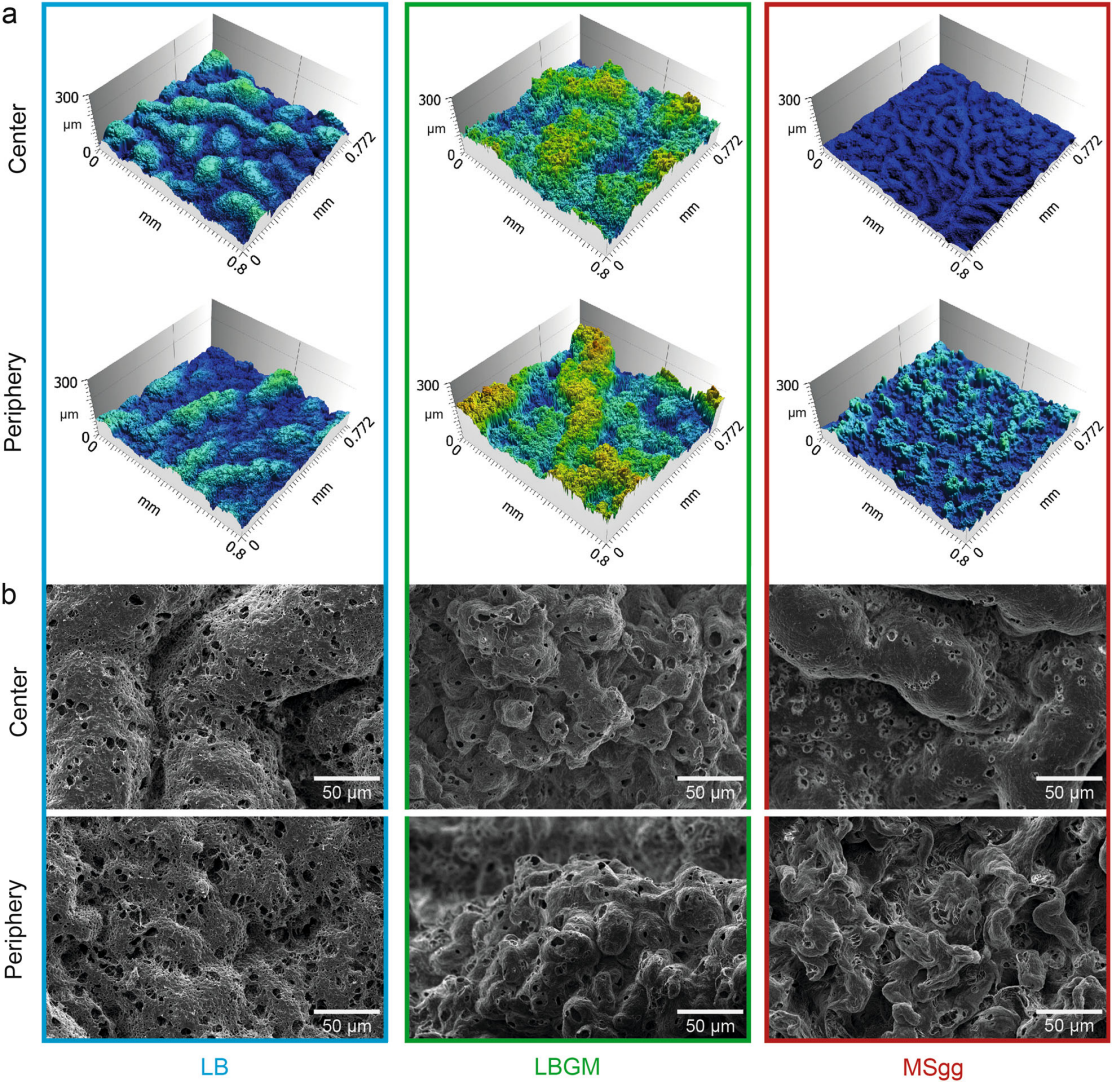
The surface topology of *B. subtilis* NCIB 3610 biofilms depends on the biofilm growth medium. When analyzed by light profilometry, *B. subtilis* NCIB 3610 biofilms show differences in their height features (**a**). Similar differences can also be observed in SEM images (**b**). Furthermore, for biofilms grown on MSgg, the topology in the center and the periphery of the colonies seems to be different

Even though the detailed wetting behavior of rose petals and lotus leaves is different, they both constitute strong hydrophobic biosurfaces. A structural feature the two biosurfaces share is that they both exhibit a rough surface topology on the microscale as well as on the nanoscale. Thus, in a next step, we test whether the peripheries of two hydrophobic biofilm variants show stronger roughness features than those of the hydrophilic biofilm variant. A suitable technique to characterize the surface topology of a material on the microscale is light profilometry. Indeed, when we analyze the surfaces of our three biofilm variants with this technique, the obtained surface profiles reveal different topologies (Fig. 2a).

The peripheral region of biofilm grown on LB agar exhibits relatively smooth height features with peaks in the range of ~ 80 µm. In contrast, the peripheral surface of the hydrophobic LBGM biofilm appears to be much rougher: Indeed, here the maximal height difference in the surface structures is on the order of ~ 290 µm. Finally, the MSgg biofilm shows peripheral surface features of ~ 160 µm height but narrower spacing than observed for LB biofilm. To confirm these differences in the surface topology of the three biofilm variants, SEM images of the biofilm samples were acquired (Fig. 2b). At low magnification, SEM probes a similar length scale as light profilometry, and indeed the visual impression obtained from the profilometry images is confirmed by the SEM pictures: the peripheral region of the hydrophilic biofilm appears to be smoother than that of the hydrophobic biofilm variants which, in turn, both show a multitude of roughness features (Fig. 2). At higher magnification, the topological difference between the hydrophilic and the hydrophobic biofilm variants is even more pronounced (Extended Data Fig. 3). In the periphery, the surface structure of the hydrophilic biofilm is highly porous. In contrast, in the peripheral surface of the hydrophobic biofilm variants, the bacteria are tightly packed and the surface shows little to no pores (Extended Data Fig. 3)

**Fig. 3:**
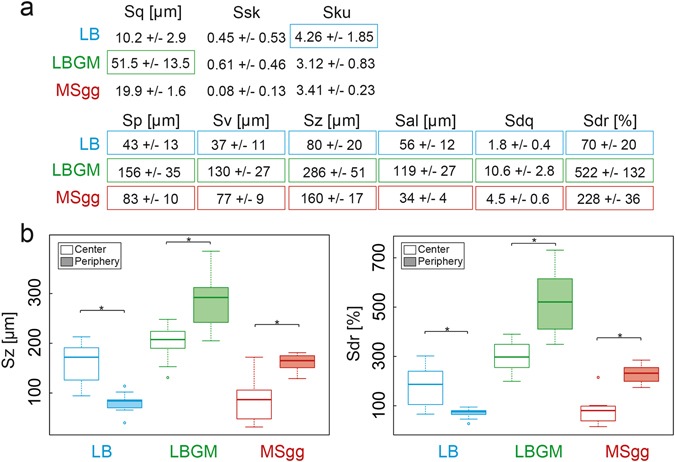
Metrological quantification of the surface topologies of *B. subtilis* NCIB 3610 biofilms. A broad range of metrological parameters **a** are calculated from the surface profiles obtained from the peripheral regions of biofilms. With a subset of those metrological parameters, both the central and peripheral regions of a given biofilm colony are compared (**b**). Boxed values in **a** and asterisks in **b** indicate statistically significant differences (*n* ≥ 9, *p* < 0.05)

In addition to those pronounced structural differences in the periphery between the hydrophilic and the hydrophobic biofilms, it appears that the two hydrophobic biofilm variants differ from each other in terms of their surface topology. Both the profilometry as well as the SEM images suggest that the spacing between the peripheral surface features on the MSgg biofilm is narrower than on the LBGM biofilm. Thus, in a next step, the profilometry images are analyzed in more detail. The idea is to calculate quantitative metrological parameters from the surface periphery of the biofilm colonies that either verify or falsify the visual impression discussed so far. The root mean squared roughness, *Sq*, is widely used to characterize the surface topology of materials. However, neither *Sq* nor higher order powers of the surface height, such as skewness *Ssk* or kurtosis *Sku* can sufficiently distinguish between the three biofilm variants (Fig. 3a). On the other hand, absolute height parameters such as the maximal peak height, *Sp*, the deepest valley depth, *Sv*, and the maximum height, *Sz*, show significant (*p* < 0.05) differences among all three biofilm surfaces. With each of those three parameters, we find the smallest feature size for the LB grown biofilm, intermediate values for MSgg biofilm, and the largest features for LBGM grown biofilm (Fig. 3a)—in full agreement with the visual impression discussed before. To quantify the spacing between individual roughness features, the length of the fastest decay of the autocorrelation function, *Sal*, is calculated. The widest spacing is shown by LBGM biofilms where we find *Sal*_LBGM_ = (119 ± 27) µm. This value also shows significant (*p* < 0.05) differences among all biofilm variants (Fig. 3a). Furthermore, the root mean square surface slope, *Sdq*, is considered—a parameter which combines both roughness as well as spacing information; for two surfaces with identical roughness values, a lower *Sdq* value indicates a texture which is spaced more widely. For the previous parameter, significant differences (*p* < 0.05) among the three biofilm variants are observed as well (Fig. 3a). Finally, the developed interfacial area, *Sdr*, is determined—which indicates the complexity of a surface by comparing the actual surface and the projected surface. Since the relative increase in the total surface area is directly related to the wetting energy,^44^ we expect also this parameter to be able to distinguish among the three biofilm variants. Indeed, the calculated *Sdr* values are significantly (*p* < 0.05) different for the three biofilms; the LB biofilm shows the smallest increase in the surface area, and we find the largest value for the LBGM biofilm (Fig. 3a).

Although we have found a number of metrological surface parameters which can successfully distinguish between the peripheries of the three biofilm types, not all surface texture parameters listed in ISO norms returned significant differences. Thus, in a next step, we ask if a more general mathematical analysis of the surface topology can be used instead of calculating a whole list of individual metrological parameter values. A discrete Fourier analysis is commonly used to analyze complex signals and to quantitatively determine the contribution of sub-signals with different wavelengths.^45^ In such an approach, the 2D surface of the biofilm is approximated by a sum of sinusoidal waves. Then, the average power spectral density lists the amplitudes of those waves as a function of the corresponding wavelengths. As depicted in (Extended Data Fig. 4), this Fourier analysis can clearly differentiate between the peripheral regions of the three biofilm variants: At small wavelengths, the hydrophilic biofilm (LB) clearly stands out, as here the amplitudes are almost one order of magnitude smaller than for the other two biofilm types (LBGM, MSgg). This regime, e.g., wavelengths in the range of tens of micrometers, is normally referred to when a surface roughness is determined. Consistently, we also found the smallest *Sq* value for the periphery of the hydrophilic LB biofilm. However, higher wavelengths contribute to the surface topology as well, and constitute a surface feature which is typically referred to as waviness. At those larger wavelengths, i.e., in the range of 100 µm and above, the LBGM biofilm has peripheral surface features with amplitudes that are approximately one order of magnitude larger than those of both the hydrophilic (LB) and the rose petal like (MSgg) biofilm. This result agrees very well with both the SEM images shown in (Fig. 2b) (in which the spacing between the individual peripheral surface features appears to be smaller for the MSgg grown biofilm than for the LBGM variant) and the *Sal* values discussed before.

To further challenge our hypothesis that the wetting behavior of biofilms is linked to differences in their surface topology, we extend our metrological analysis and study the spatial heterogeneity of the biofilm surfaces. When the central and peripheral regions of a given bacterial biofilm colony are compared, a similar relationship between the surface topology and the wetting resistance of those biofilm regions is observed (Fig. 3b) as discussed before, when we compared the peripheral regions of the three biofilm variants (Fig. 3a). For instance, the central (hydrophilic) area of the MSgg biofilm displays a smoother surface than its (hydrophobic) periphery (Fig. 2). Quantitatively, this is reflected in the significantly (*p* < 0.05) lower *Sz* and *Sdr* values calculated for the biofilm center (Fig. 3b). In contrast, the central and peripheral region of LBGM grown biofilm both exhibit hydrophobic properties. Consistently, higher *Sz* and *Sdr* values are obtained from the local surface profiles at both locations of this biofilm than for the MSgg or LB biofilms (Fig. 3b). Finally, the central and the peripheral regions of LB biofilm colonies show similar hydrophilic wetting behavior and *Sz* and *Sdr* values mostly lower than those obtained in hydrophobic biofilm areas (Fig. 3b). This extended analysis confirms our notion that the surface topology of the biofilms and their wetting behavior are directly related.

### Physical wetting regimes on the different *B. subtilis* NCIB 3610 biofilms

So far, we have argued that the differences in the surface structure of *B. subtilis* NCIB 3610 biofilms are correlated with the observed differences in their wetting behavior. Quantitative metrological parameters calculated from the surface topography of the biofilms support this idea. Moreover, the two hydrophobic biofilm variants showed significantly different surface topologies as well. Our wetting experiments revealed a lotus leaf-like wetting resistance for LBGM biofilms and a rose petal-like wetting resistance for the periphery of MSgg biofilms. Thus, we next ask if this analogy can be extended, i.e., if similar physical wetting mechanisms as described for the corresponding leaf/petal surfaces are also responsible for the wetting resistance of the hydrophobic biofilms. For instance, trapped air bubbles are reported to locally separate the microscopic surface features of a lotus leaf and a water droplet.^15^ This mechanism is referred to as a Cassie/Baxter state, a three-phase wetting interface comprising a solid, a liquid, and an air component.^16^ In contrast, for rose petals an impregnated Cassie regime is reported, i.e., the microstructures of the rose petal surface are in contact with the wetting fluid.^43^ High adhesion forces towards a water droplet and a pronounced contact angle hysteresis—as also observed for the MSgg biofilm—are a direct consequence of this impregnated wetting state.

**Fig. 4:**
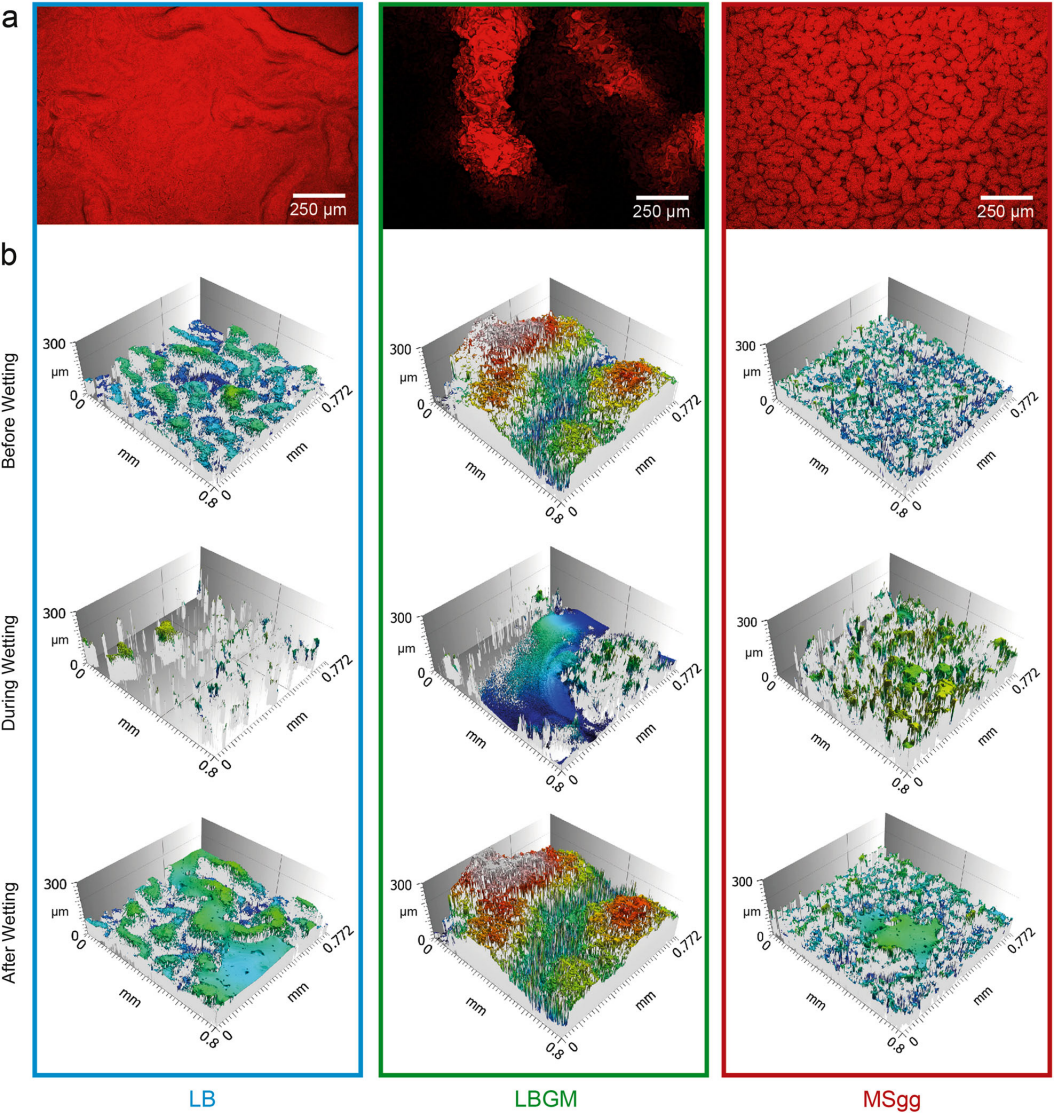
Surface analysis of the peripheral region of biofilms before, during, and after wetting. Confocal fluorescence images **a** show differences in biofilm staining after local wetting with a dying solution. Light profilometry images before, during, and after wetting **b** suggest a homogenously wetted surface for LB biofilms, a three-phase Cassie–Baxter wetting regime for LBGM biofilms, and an impregnated Cassie wetting regime for MSgg biofilms (see main text for details). Please note the much higher resolution of the images in *z*-direction than in *x*-direction and *y*-direction

To test whether the two hydrophobic biofilm variants can be described by a Cassie/Baxter and an impregnated Cassie wetting state, respectively, we next evaluate the surface-liquid interface for the three biofilm variants. In a first step, we bring the biofilms in contact with an aqueous staining solution and then image the biofilm surfaces using fluorescence microscopy. Z-projections of confocal image stacks (Fig. 4a) show differences in the staining behavior of the biofilms, which are consistent with the differences in the surface topologies and the different wetting regimes discussed before: biofilm grown on LB agar is stained uniformly as expected for a hydrophilic surface. Biofilm grown on LBGM agar is mainly stained at the peak areas of the surface structures, which suggests that the aqueous staining solution did not get in contact with the valleys of the biofilm surface. In contrast, MSgg biofilm seems to be stained much more efficiently than the LBGM biofilm, as we only find thin non-fluorescent valleys separating the well-stained surface roughness features. Apparently, the MSgg biofilm variant—although showing hydrophobic behavior in its periphery-allows most of its surface to be wetted by the staining solution. In contrast, LBGM biofilm surfaces seem to partially avoid contact with water.

Profilometry images acquired before, during and after wetting of the biofilm colonies with a dye-free water droplet (Fig. 4b) support the results obtained from biofilm staining. For LBGM biofilm, areas with a large flat interface appear during wetting. When the water droplet is removed with compressed air, these flat interfacial areas disappear again, and the identical biofilm surface topology is found as it was present before wetting. This result suggests that the flat interfaces observed during the wetting process are established by trapped air bubbles separating the rough biofilm surface and the bottom of the water droplet.

In contrast, for the peripheral region of the biofilm grown on MSgg agar, the surface topology of the biofilm/water interface appears to be similar to the biofilm/air interface imaged before wetting (Fig. 4b). Here, planar interfacial areas as observed for the lotus leaf-like LBGM biofilm do not occur. However, after the water droplet has been removed with pressurized air, we do detect small interfacial areas with a flat topology. Those flat interfaces are established at higher *z*-coordinates than the biofilm contour. Thus, they likely represent the upper surface of micro-cavities filled with water. This interpretation would also be consistent with the idea that—due to the presence of an impregnated Cassie state—the MSgg biofilm exhibits strong adhesion forces towards water droplets. Of course, also the LB biofilm surface exhibits residual water after the wetting process, but here this finding is not surprising considering that a Wenzel wetting state^29^ is expected for a hydrophilic material.

### Biochemical composition of the different *B. subtilis* NCIB 3610 biofilms

**Fig. 5:**
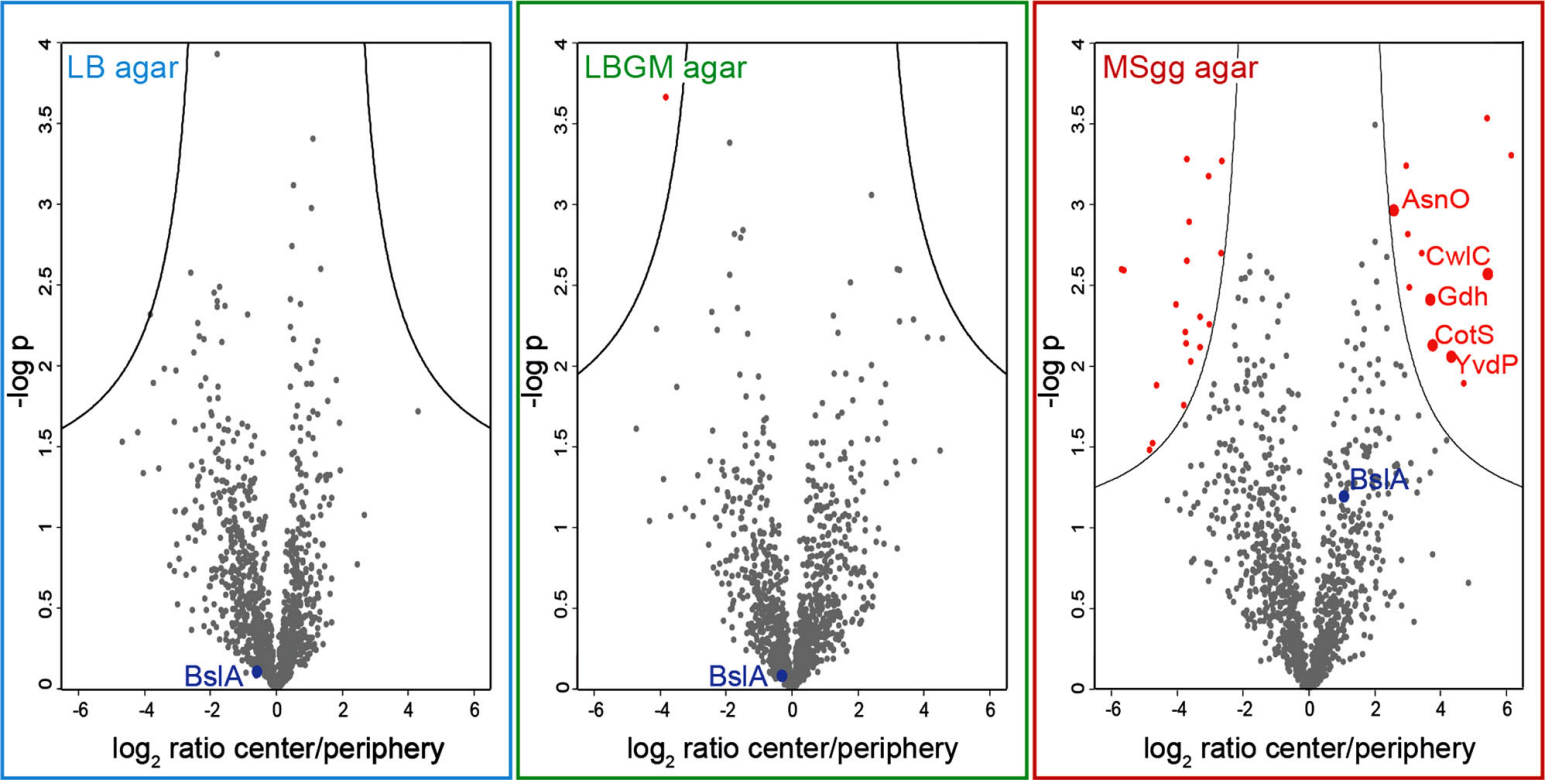
Proteomics analysis of the center and periphery of *B. subtilis* NCIB 3610 biofilms grown on different agar substrates. For statistical evaluation of the biofilm composition, volcano plots are generated from data of three independent experimental replicates to illustrate differences in protein expression between the center and periphery of the different biofilm types, i.e., LB, LBGM, and MSgg biofilm. The *y*-axis represents the *p*-value and the *x*-axis lists the binary logarithm of the n-fold change in protein expression levels between the center and the periphery of a biofilm colony. The *solid lines* indicate a significance level of *p* = 0.05 and a required minimum fold change of 2 (s0 = 1) which is used as a cut-off for significance. The *dots* below the *cut off lines* correspond to proteins expressed both in the center and periphery without significant differences. The *red dots* above the *cut off lines* represent proteins which are expressed at significantly higher or lower levels in the center compared to the periphery of that colony

Having demonstrated that the three biofilm variants indeed exhibit different physical wetting mechanisms, we ask in a last step whether the observed differences in biofilm wetting and topology are accompanied by differences in the biofilm composition. This assumption is reasonable considering that a nutrient rich medium such as LB agar and a minimal growth medium such as MSgg agar is likely to give rise to different proteomic expression profiles of the bacteria. Indeed, a mass spectrometry (MS) based analysis of extracellular proteins of the peripheral regions of LB, LBGM, and MSgg biofilms reveals significant differences in protein expression (Extended Data Fig. 5). Significant alterations in the expression level of proteins are also detected between the central and peripheral region of MSgg biofilm colonies, but not when comparing the center and peripheries of LB or LBGM biofilm colonies, respectively (Fig. 5). This finding is especially interesting as only the MSgg biofilm variant showed spatially heterogeneous wetting behavior. Moreover, among those proteins expressed at higher levels in the central region of these MSgg biofilms, mostly such proteins which are related to spore formation, are overrepresented (Extended Data Table 2). Indeed, we detect a large number of bacterial spores in the center of MSgg biofilm colonies, but not in the periphery (Extended Data Fig. 6). The occurrence of spores is consistent with the limited amount of nutrients present in MSgg agar.

However, the surface layer protein BslA (= YuaB) which is suggested to contribute to the hydrophobic properties of *B. subtilis* NCIB 3610 biofilms^27, 40^ is not detected at significantly different levels in either region of the MSgg biofilm. When we analyze the wetting behavior of a biofilm colony generated by a *B. subtilis* mutant strain that is unable to produce BslA,^27^ we observe strongly hydrophilic colonies on all agar variants (Extended Data Fig. 7). Consistently, all those colonies formed by the mutant strain show smooth surface topologies with *Sz* and *Sdr* values comparable to (or even lower than) those obtained for hydrophilic wild type colonies. Moreover, in a previous study performed by Kobayashi et al.,^27^ it was observed that a *B. subtilis* mutant strain unable to produce the fiber forming protein tasA generates hydrophilic biofilm colonies although BslA is present. Together, those findings underscore that the wetting behavior of *B. subtilis* biofilms is also strongly influenced by the topology of the biofilm and not only by the presence or absence of certain hydrophobic surface layers formed by proteins or other secreted biomolecules. Of course, our proteomics analysis does not test for other classes of biomolecules beyond polypeptides (such as lipids, DNA, or metabolic byproducts), yet the presence or absence of such other biofilm macromolecules may also have an impact on the topology of the biofilm colony. Because of that, it is not trivial to disentangle the contribution of a specific biofilm component on the chemical properties of a biofilm surface and its topology—and both parameters contribute to the wetting resistance of the biofilm.

## Conclusions

In summary, with the metrological approach introduced here, we were able to correlate the topologies of bacterial biofilms formed by *B. subtilis* NCIB 3610 with the wetting behavior of those bio-surfaces. We have observed different surface topologies not only for biofilms grown on different agar variants, but also within a given biofilm colony grown under limited nutrient conditions. In all cases where we observed differences in the biofilm wetting behavior, those differences were accompanied both with significant topological differences as well as alterations in the protein composition of the biofilm matrix.

The identical trend, i.e. hydrophilic behavior on LB agar, hydrophobic (lotus-like) behavior on LBGM agar and hydrophobic (rose-petal like) behavior on MSgg agar, is observed for the peripheral regions of *B. subtilis natto* biofilms and also correlates with similar differences in the biofilm topologies (Extended Data Fig. 8). Interestingly, the *Sdr* values we obtain for the peripheral region of the MSgg biofilm variants of both *B. subtilis* NCIB 3610 and *B. subtilis natto* showing rose petal-like wetting resistance agree very well with the values we obtain for actual rose petals (Extended Data Fig. 9). This further underscores the analogy drawn between the wetting resistance of rose petals and that of hydrophobic MSgg biofilm.

From a biological point of view, the existence of three different wetting regimes for *B. subtilis* biofilms is curious. Whereas a hydrophilic biofilm surface might not be ideal as prolonged contact with water will facilitate biofilm dissolution and erosion over time,^46^ it is less obvious why biofilms would exhibit two variants of hydrophobic behavior. At this point, it might be important to recall that we observed an impregnated Cassie state (i.e., rose petal-like wetting) for biofilms grown during limited nutrient supply (i.e., on MSgg agar). Here, in contrast to the lotus-like state, where air bubbles separate the biofilm surface and the water phase, the biofilm surface is in contact with water but still behaves hydrophobic. We speculate that this particular wetting state could be helpful for two reasons: first, the impregnated hydrophobic surface may help avoid biofilm erosion while maintaining a moist biofilm surface which, in turn, would prevent the biofilm from drying. At the same time, small water droplets on the biofilm surface could create a microenvironment which allows the biofilm bacteria to spread by swimming or flagellum-independent migration^47^—thus enabling them to explore neighboring areas in search of additional nutrients.^48^ When the nutrient supply becomes limiting, the presence of dead cells in the biofilm could be one possible factor contributing to the occurrence of a rough biofilm topology,^49^ which—as we demonstrate here—alters the wetting behavior of the biofilm. It appears reasonable that the growth of biofilms both in nutrient-rich and nutrient-poor environments has led to the development of different wetting properties of biofilm colonies which are adapted to the particular environmental conditions.

A spatially heterogeneous wetting behavior as we observed it on MSgg grown biofilms has already been reported for certain plants^50, 51^ and animals,^52, 53^ where it is thought to promote water collection. For instance, arid climate plants such as *Lupin regalis* possess leaves with hydrophobic tips, but—at the same time—a highly hydrophilic inner region.^50^ This enables the plant to ‘catch’ water droplets from rain or dew on their leaves until they are big enough to roll into the center and then down to the stem and the roots. Such a natural water guidance mechanism based on surface polarity has inspired the design of artificial structures that manipulate water flux.^54–56^ A similar mechanism may aid biofilms growing in limiting nutrient conditions (as they are also present in MSgg agar) to guide water towards the center of the colony—potentially to gather more nutrients from the surroundings or to cause osmotic spreading of the bacteria and thus reach a larger area of nutrient availability.

Of course, when dealing with biofilms growing in pipes or on catheters, their hydrophobic surface properties constitute a serious issue: only when an aqueous solution containing anti-bacterial agents is in contact with the biofilm surface, the bactericidal molecules will have a chance to enter the biofilm. Therefore, finding a method to convert the wetting resistance of biofilms from lotus-like to rose petal-like could be an important stepping stone in fighting biofilms. This clearly demonstrates the need to better understand why bacteria generate different biofilm topologies on different surfaces and how to exert control over this process. Together with the development of dedicated anti-fouling surfaces that prevent bacterial adhesion and therefore the initiation of biofilm formation,^57–59^ improving the accessibility of biofilm surfaces to liquids is an important goal. Success in this particular problem will also allow for more efficient weakening of the mechanical properties of biofilms, e.g., by targeting the bacterial adhesion machinery^60, 61^ thus facilitating biofilm removal.

## Methods

### Biofilm cultivation

*B. subtilis* NCIB 3610 was obtained from the lab of Roberto Kolter. A liquid culture was prepared overnight as follows: 15 mL of sterile 2.5% Luria/Miller LB medium (Carl–Roth, Karlsruhe, Germany) were inoculated with a frozen bacterial/glycerol stock. Then, the bacterial solution was incubated at 37 °C at 90 r.p.m. in a shaking incubator (Sartorius, Goettingen, Germany) overnight. Three different growth media were used: first, standard 2.5% Luria/Miller LB medium; second, LBGM medium, i.e., 2.5% LB medium enriched with 100 µM Manganese(II)sulfate (MnSO4), and 1% glycerol; third, MSgg minimal medium containing 5 mM potassium phosphate, 100 mM Mops, 2 mM MgCl2, 700 µM CaCl2, 50 µM MnCl2, 50 µM FeCl3, 1 µM ZnCl2, 2 µM thiamine, 0.5% glycerol, 0.5% glutamate, 50 µg/mL tryptophan, 50 µg/ml phenylalanine, and 50 µg/mL threonine (adapted from Branda et al.).^41^ To generate solid nutrient layers for biofilm growth, the different culture media were mixed with 1.5 % Agar-Agar (Carl–Roth, Karlsruhe, Germany), autoclaved and the still hot liquid was poured into petri dishes. To obtain bacterial biofilm colonies, three separate 5 µL drops of bacterial liquid culture were pipetted onto each petri dish and cultured at 37 °C for 24 h. For simplicity, the biofilms grown on the different agar variants are referred to as “LB biofilm”, “LBGM biofilm,” and “MSgg biofilm” in the main text.

### Light profilometry

Profilometry images are acquired using a Nanofocus µsurf profilometer (NanoFocus AG, Oberhausen, Germany). Images are taken from bacterial colonies with 20× magnification resulting in surface images with an area of 800×772 µm. Missing data points were interpolated and the scanned area was then evaluated with the software µsoft (Version 6.0, NanoFocus AG, Oberhausen, Germany).

With this software, the following parameters are calculated for surface characterization: the root mean square surface roughness ${{Sq}}{\rm{ = }}\sqrt {\frac{1}{A}{\int} {{\int}_A {{z^2}} } \left( {x,y} \right)dxdy}$, the skewness of the height distribution ${{Ssk}}{\rm{ = }}\frac{1}{{{S_q}^3}}\left[ {\frac{1}{A}{\int} {{\int}_A {{z^3}} } \left( {x,y} \right)dxdy} \right]$, the kurtosis of the height distribution ${{Sku}}{\rm{ = }}\frac{1}{{S{q^4}}}\left[ {\frac{1}{A}{\int} {{\int}_A {{z^4}} } \left( {x,y} \right)dxdy} \right]$, the maximum peak height *Sp*, maximum pit height *Sv*, the maximum height ${{Sz}}\,{\rm{ = }}\,Sp + Sv$, the developed interfacial area ratio, ${{Sdr}}{\rm{ = }}\frac{1}{A}\left[ {{\int} {{\int}_A {\left( {\sqrt {\left[ {1 + {{\left( {\frac{{\partial z\left( {x,y} \right)}}{{\partial x}}} \right)}^2} + {{\left( {\frac{{\partial z\left( {x,y} \right)}}{{\partial y}}} \right)}^2}} \right]} - 1} \right)} } dxdy} \right]$, the root mean square gradient ${{Sdq}}{\rm{ = }}\sqrt {\frac{1}{A}{\int} {{\int}_A {\left[ {{{\left( {\frac{{\partial z(x,y)}}{{\partial x}}} \right)}^2} + {{\left( {\frac{{\partial z(x,y)}}{{\partial y}}} \right)}^2}} \right]} } dxdy}$, and the autocorrelation length ${{Sal}}{\rm{ = }}\mathop {{\min }}\limits_{tx,ty \in R} \sqrt {t{x^2} + t{y^2}}$, where $R{\rm{ = }}\left\{ {\left( {tx,ty} \right):ACF\left( {tx,ty} \right) \le 0.2} \right\}$ and *ACF* denotes the autocorrelation function. All the parameters are defined in the ISO 25178 norm.

The software R together with the user interface RStudio were used for statistical analysis of the surface parameters obtained from profilometry. The exact number of biological replicates is given in the caption of each figure. The obtained data distributions are first checked for normality by Q-Q plots and a Shapiro–Wilk test, and homogeneity of variances is confirmed by a Levene test. To detect significant differences between the examined groups, one-way ANOVAs, and for pairwise comparisons Tukey post-hoc tests were carried out. A *p*-value of *p* < 0.05 was used to determine significant differences between data sets.

For imaging the biofilm/water droplet interface, we scanned vertically through the sample starting at the upper surface of the water droplet. While moving the focus plane downwards, a second interface appeared. This is the interface shown in (Fig. 4b). A fair amount of valid data points can be acquired at the biofilm/air interface. However, for the solid/liquid (biofilm/water) interface, only a small amount of valid data points can be acquired. We assume this is caused by the high water content of bacterial biofilms which renders the biofilm/water interface difficult to image with light profilometry.

The much higher resolution of the topological images in *z*-direction compared to *x*- and *y*-direction is due to the fact that the profilometer defines the interface position as follows: the profilometer detects reflected light. For each *x*/*y*-position, the profilometer lens is moved over a certain range in *z*-coordinates and the intensity of reflected light is measured for each of those *z*-positions. Typically, a Gaussian distribution is obtained, and the peak in reflected light intensity corresponds to the *z*-position of the surface, since here the reflected light intensity is maximal (due to the confocal configuration of the microscopy setup).

### Confocal fluorescence microscopy

For biofilm staining, a droplet of a dying solution (10 µg/mL Atto 488 dissolved in PBS) is placed onto the biofilm. After an incubation time of 1 minute, the droplet is removed by pressurized air, and the wetted region is imaged with a Leica TC S SP5 confocal fluorescence microscope. A 10× objective and 100 Hz scanning speed was used for image acquisition and an area of 1024 × 1024 px was scanned per image. The images shown in (Fig. 4a) are *z*-projections of a *z*-stack containing 25 individual scans.

### Scanning electron microscopy

For SEM images, biofilm samples were shock-frozen with liquid nitrogen and then dried by lyophilization for at least 48 hours. Light profilometry confirmed that this freezing/lyophilization process did not significantly alter the biofilm microtopology. The lyophilized biofilm layers were placed onto the aluminum SEM sample holders and sputtered for 40 seconds with Au (MED 020, BAL-TEC, Balzers, Liechtenstein). The SEM (JEOL-JSM-6060LV, Jeol, Eching, Germany) was operated at an acceleration voltage of 5 kV.

### Mass spectrometry sample preparation, measurement, and analysis

Proteomics analysis of biofilm samples was performed in independent triplicates. Bacteria (37 mg replicate 1, 24 mg replicates 2 and 3) from the center and periphery of biofilms grown on different agars (LB, LBGM, and MSgg) were resuspended in 0.9 % NaCl. Extracellular proteins were extracted from biofilms by subsequent vortexing and centrifugation (20 min, 12000 g, 5 °C) for three times, supernatants were pooled and intact bacteria removed by filtering with 0.45 and 0.22 µm filters (Millipore). Absence of bacterial growth was checked by platting aliquots on LB agar. Components smaller 3 kDa were removed (filters: modified PES, 3 kDa, VWR) and proteins precipitated with chloroform/methanol according to Wessel–Flügge. Proteins were solubilized in 7M Urea/2M thiourea, reduced, alkylated and enzymatically digested with LysC and trypsin. Generated peptides were desalted on C18 material, lyophilized and resolved in 0.1 % formic acid for MS measurement. MS analysis was performed on an Orbitrap Fusion instrument coupled online to an Ultimate 3000 Nano HPLC via an electrospray easy source (Thermo Fisher Scientific). Peptides were separated on a 50 cm C18 column (particles 2 µm, 100A, inner diameter 75 µm, Thermo Fisher Scientific) constantly heated at 50 °C. The gradient was run from 5–32 % acetonitrile, 0.1 % formic acid during a 152 min method (7 min 5%, 105 min to 22%, 10 min to 32 %, 10 min to 90 %, 10 min wash at 90 %, 10 min equilibration at 5%) at a flow rate of 300 nL/min. Most intense ions from survey scans measured in the orbitrap (*m*/*z *300–1500) were chosen for fragmentation with high-energy collisional dissociation and spectra acquired in the ion trap (max injection time 35 ms, target value 1e4) while the instrument was operated in top speed mode. The search for MS/MS based peptide identification was performed with Max Quant (version 1.5.3.8)^62^ against the *B. subtilis* 168 UniProtKB database (July 2016). Default settings were used with the following exceptions: minimal number of unique peptides for protein identification was set to two, fast label-free quantification and match between runs options were enabled. Statistical analysis was performed in Perseus (as part of the MaxQuant environment).^63^ Only proteins that were identified based on at least 2 MS/MS counts and valid ratios in all three replicates of either of the six states were considered for data analysis. Missing values were then imputed on the basis of a normal distribution (width = 0.3, down-shift = 1.8). Volcano plots were generated on the basis of a two-sample *t*-test (both sides, FDR = 0.05, S0 = 1). Overrepresentation analysis is based on gene ontology annotations and was performed with the Bingo App in the Cytoscape environment.^64^ Statistically significant regulated proteins from the volcano plot were compared to all proteins present in the plot in the category of biological process. Analysis was based on a hypergeometrical test with the multiple testing correction according to Benjamini Hochberg and a significance level of 0.05.

## Electronic supplementary material

Extended data

## Electronic supplementary material

Supplementary Information accompanies the paper on the *npj Biofilms and Microbiomes* website (10.1038/s41522-017-0018-1).
