# Supplementary material for: Surface topology affects wetting behavior of Bacillus subtilis biofilms
Source: NPJ Biofilms Microbiomes. 2017 Apr 25;3:11. doi: 10.1038/s41522-017-0018-1 (PMC5460217; doi:10.1038/s41522-017-0018-1)
Supplement: Supplementary file 1 — Extended data [file 41522_2017_18_MOESM1_ESM.pdf]

Extended data for

Surface topology affects wetting behavior of *Bacillus subtilis* biofilms

Moritz Werb[1,*], Carolina Falcón García[1,*], Nina C. Bach[2], Stefan Grumbein[1], Stephan A. Sieber[2], Madeleine Opitz[3] and Oliver Lieleg[1,#]

[1] Department of Mechanical Engineering and Munich School of Bioengineering, Technische Universität München, Garching, Germany

[2] Department of Chemistry, Technische Universität München, Garching, Germany

[3] Center for NanoScience, Faculty of Physics, Ludwig-Maximilians-Universität München, München, Germany

* These authors contributed equally to this work

# To whom correspondence should be addressed at oliver.lieleg@tum.de

Boltzmannstr. 11, 85748 Garching bei München, Germany. Phone: +49 89 289 10952

Extended Data Table 1 | Wetting resistance of the peripheral regions of LBGM and MSgg biofilm.

	contact angle [°] on	
	LBGM biofilm	MSgg biofilm
50% Acetone	120.6 ± 7.9	126.5 ± 5.0
50% Ethanol	130.6 ± 9.6	135.6 ± 7.3
50% Isopropanol	118.1 ± 5.6	129.7 ± 3.8
50% Methanol	129.0 ± 6.6	132.5 ± 2.9

Errors denote the standard deviation as determined from at least 3 different measurements.

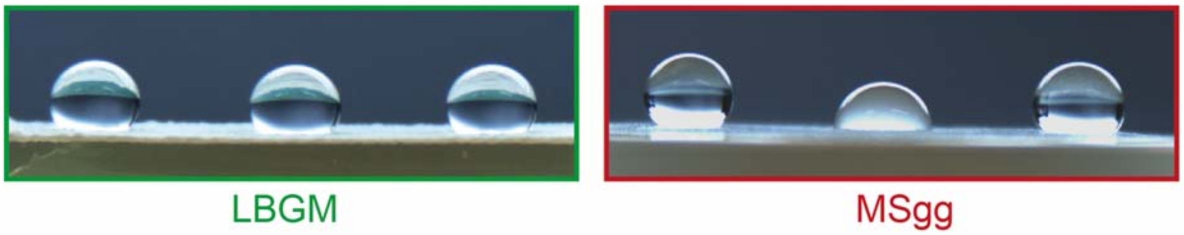

Extended Data Fig. 1 | Side view of bacterial colonies of *B. subtilis biofilm* colonies wetted with water droplets. For colonies grown on LBGM medium (left), both the central and the peripheral region of the biofilm colony show hydrophobic behavior. In contrast, biofilm colonies grown on MSgg medium (right) are spatially heterogeneous showing hydrophilic behavior in the central area and hydrophobic behavior in the periphery.

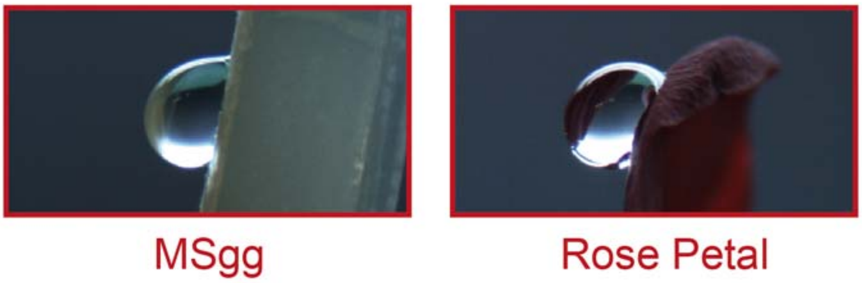

Extended Data Fig. 2 | Hanging water drop on a vertically tilted *B. subtilis* biofilm grown on MSgg agar and hanging water drop on the surface of a red rose petal. Both biosurfaces have hydrophobic properties but, at the same time, exhibit a strong adhesion force for water droplets. As a consequence, a water droplet does not roll off when the surface is tilted.

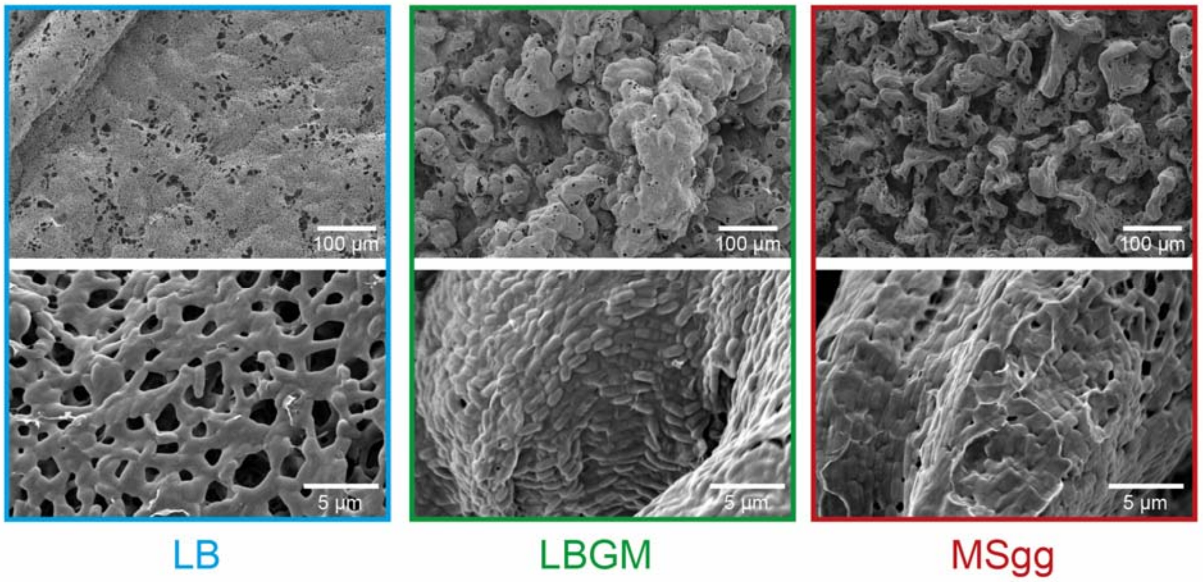

Extended Data Fig. 3 | Surface microtopology of the peripheral region of biofilms. SEM images obtained at 200x magnification (upper row) show similar surface characteristics as reported by profilometry. At 5000x magnification (lower row), the biofilm grown on LB shows a porous structure whereas, for LBGM and MSgg, the bacteria in the surface zone of the biofilm appear to be tightly packed and the biofilm exhibits little to no pores.

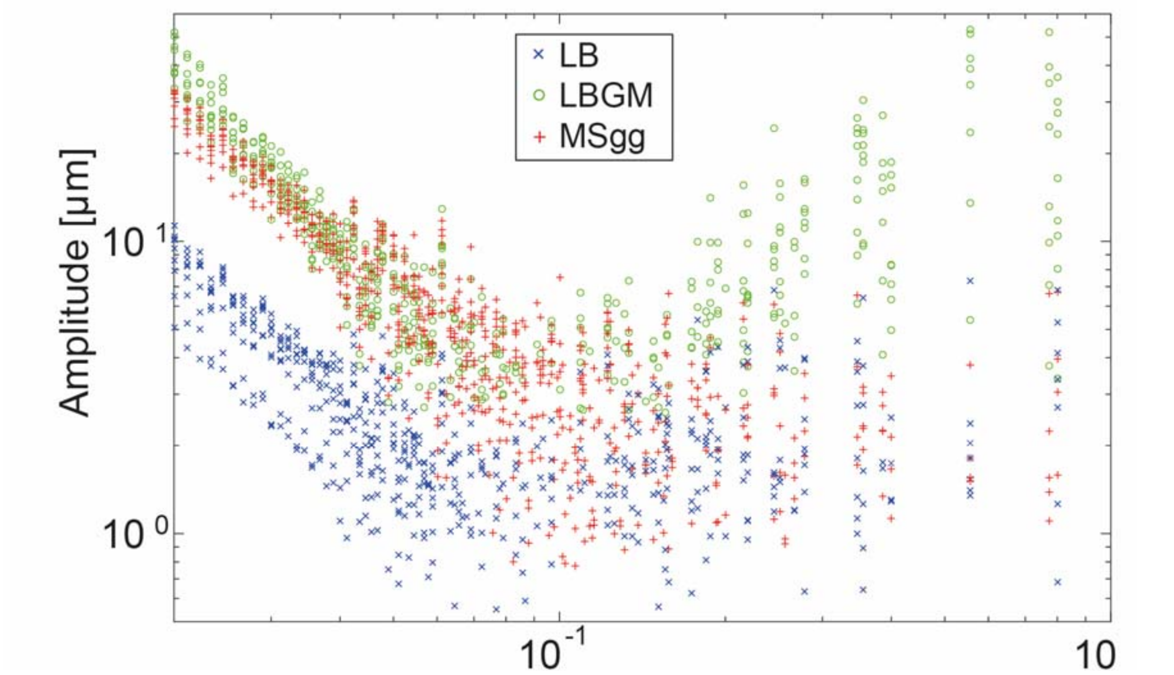

Extended Data Fig. 4 | Fourier analysis of the peripheral regions of biofilm surfaces. The average power spectral density is calculated using a fast Fourier transformation of the surface profiles. For the interpretation of the obtained data, it is crucial to recall that the spatial resolution of the profilometry images, i.e. the pixel width of the scanning process, limits the spectrum of wavelengths. As the pixel size in the analyzed profilometry images is 1.566 µm and errors for remodeling the edges of the surface are especially high for low wavelengths, wavelengths smaller than 20 µm are disregarded in the calculated power spectrum. Amplitudes at low wavelengths are one order of magnitude smaller for hydrophilic (LB medium) biofilm compared to hydrophobic (LBGM and MSgg) biofilms. For long wavelengths, ~10x higher amplitudes can be found for the biofilm grown on LBGM than for the other two biofilm variants.

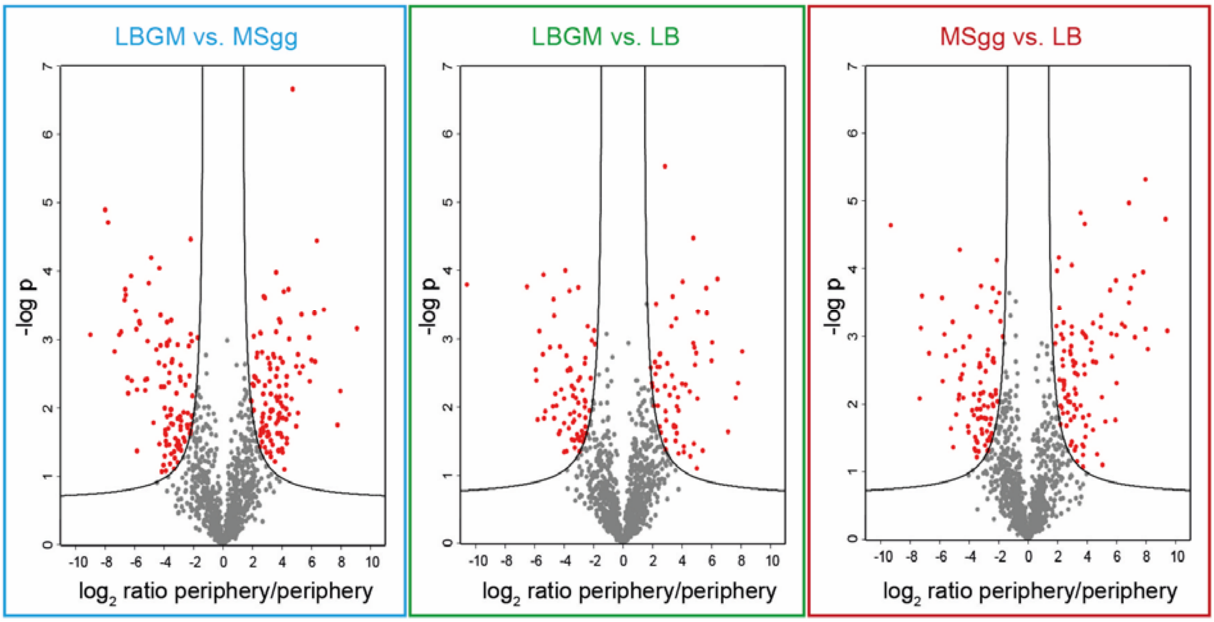

Extended Data Fig. 5 | Proteomics analysis of the peripheral regions of *B. subtilis* NCIB 3610 biofilms grown on different agar substrates. For statistical evaluation of the biofilm composition, volcano plots are generated from data of three different experimental replicates to illustrate differences in protein expression between peripheral regions of the different biofilm types, i.e., LB, LBGM and MSgg biofilm. The y-axis represents the *p*-value and the x-axis lists the binary logarithm of the n-fold change in protein expression levels between the peripheral regions of two biofilm variants. The solid lines indicate a significance level of $p = 0.05$ and a minimum fold change of 2 (s0 = 1) which is used as a cut-off for significance. The dots below the cut off lines correspond to proteins expressed in the peripheral regions of both compared colonies without significant differences. The red dots above the cut off lines represent proteins which are expressed at significantly higher or lower levels in the periphery of one colony compared to the periphery of another colony.

Extended Data Table 2 | Biological function of the overrepresented proteins involved in sporulation that were identified in Fig. 5 of the main paper

Protein	Name	Function
AsnO	Asparagine synthase (glutamine-hydrolyzing) 3	Asparagine synthase involved in a subpathway that synthesizes L-asparagine from L-aspartate; expressed late in sporulation; strains lacking AsnO fail to sporulate[65]
BslA (YuaB)	biofilm surface layer protein A	Inhibitor of KinA autophosphorylation, required for complex colony architecture[66], one component that facilitates assembly of biofilm formation[39] and is proposed to contribute to their surface repellency[27,67]
CotS	Spore coat protein S	Spore coat protein localized to the inner coat and/or on the outside of the cortex of the mature spore[68]
CotQ (YvdP)	Spore coat protein Q	Spore coat protein with oxidoreductase activity
CwlC	Sporulation-specific N-acetylmuramoyl-L-alanine amidase	Amidase that lyzes the mother cell wall at the end of sporulation by hydrolyzing the link between N-acetyl-muramoyl residues and L-amino acid residues in certain cell-wall glycopeptides[69,70]
Gdh	Glucose 1-dehydrogenase	Oxidoreductase involved in sporulation; catalytic activity: D-glucose + NAD(P)+ = D-glucono-1,5-lactone + NAD(P)H

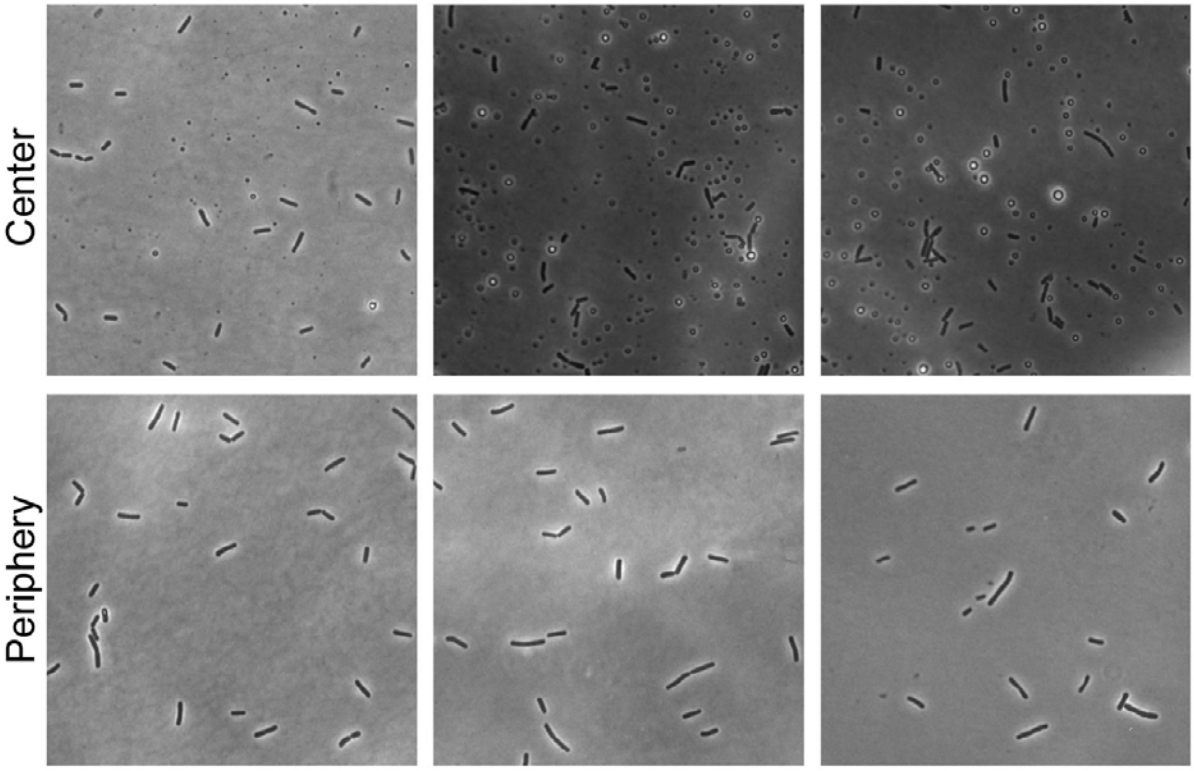

Extended data Fig. 6 | Phase contrast microscopy images of bacterial cells/spores obtained from the central and peripheral regions of *B. subtilis* NCIB 3610 biofilms grown on MSgg agar. Samples from the center and periphery of a biofilm colony were treated with bead bashing and then diluted in liquid LB media. The images were acquired with a 100x lens. In the images obtained from the center of the colonies, lots of spores are visible. However, spores are virtually absent in the samples obtained from the periphery of the colony.

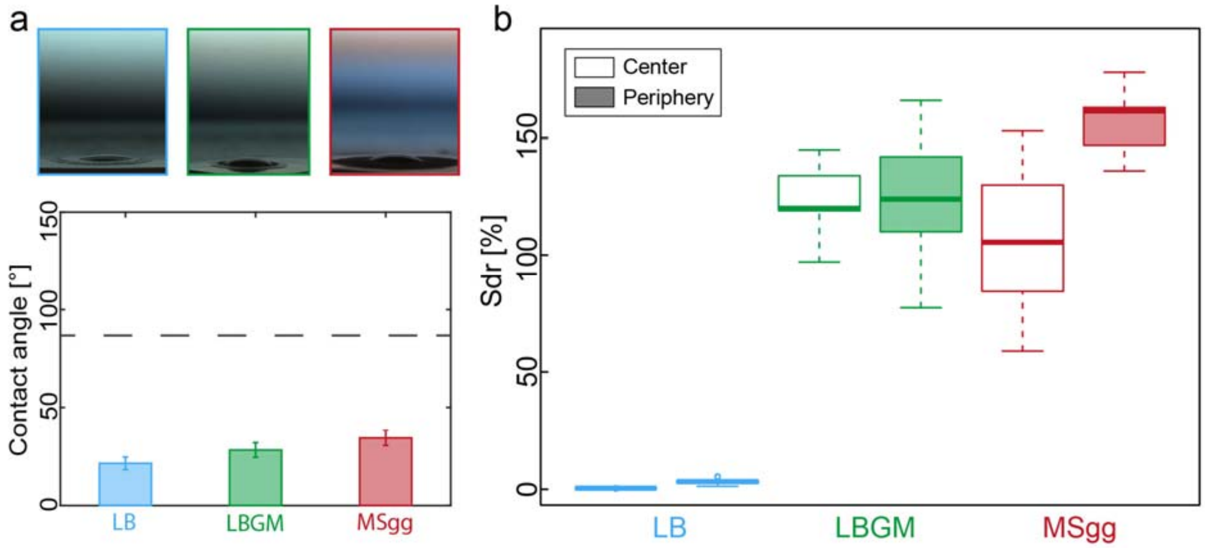

Extended data Fig. 7 | Wetting behavior and surface topology of biofilm colonies generated by the *B. subtilis* N24 mutant strain unable to produce BslA. a, When grown on LB agar, LBGM agar or MSgg agar, the biofilm colonies are very hydrophilic in either case and the measured contact angles are below 50° (n = 5) **b**, At the same time, smooth surfaces with low *Sdr* values are observed both in the center and in the periphery of the biofilm colonies ($n \geq 5$). The strain N24 was obtained from the lab of K. Kobayashi.

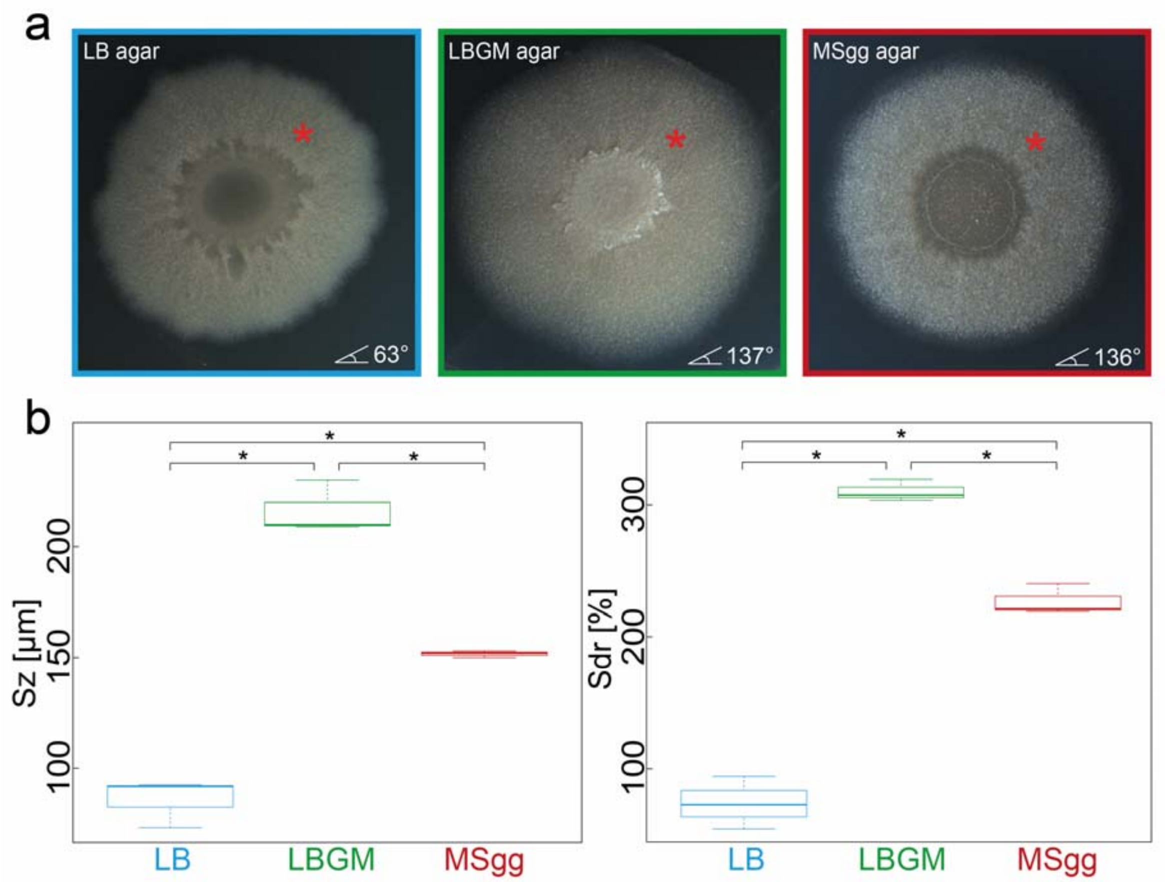

Extended Data Fig. 8 | The wetting behavior of *B. subtilis natto* biofilms depends on the growth medium and correlates with differences in the biofilm surface topology. **a**, When *B. subtilis natto* is grown on agar enriched with different molecules, the morphology of the formed macrocolonies changes, and a different wetting behavior of the biofilm peripheries is observed. The regions on the biofilm surface where the wetting tests were performed are marked with a star. **b**, A quantification of the biofilm topology obtained with light profilometry returns significant differences similar to what is shown in Fig. 3b of the main paper for *B. subtilis* NCIB 3610 biofilms. Asterisks denote significant differences ($n = 3$, $p < 0.05$). The strain *B. subtilis natto* (strain 27E3) was purchased from the Bacillus Genetic Stock Center (Ohio State University, USA).

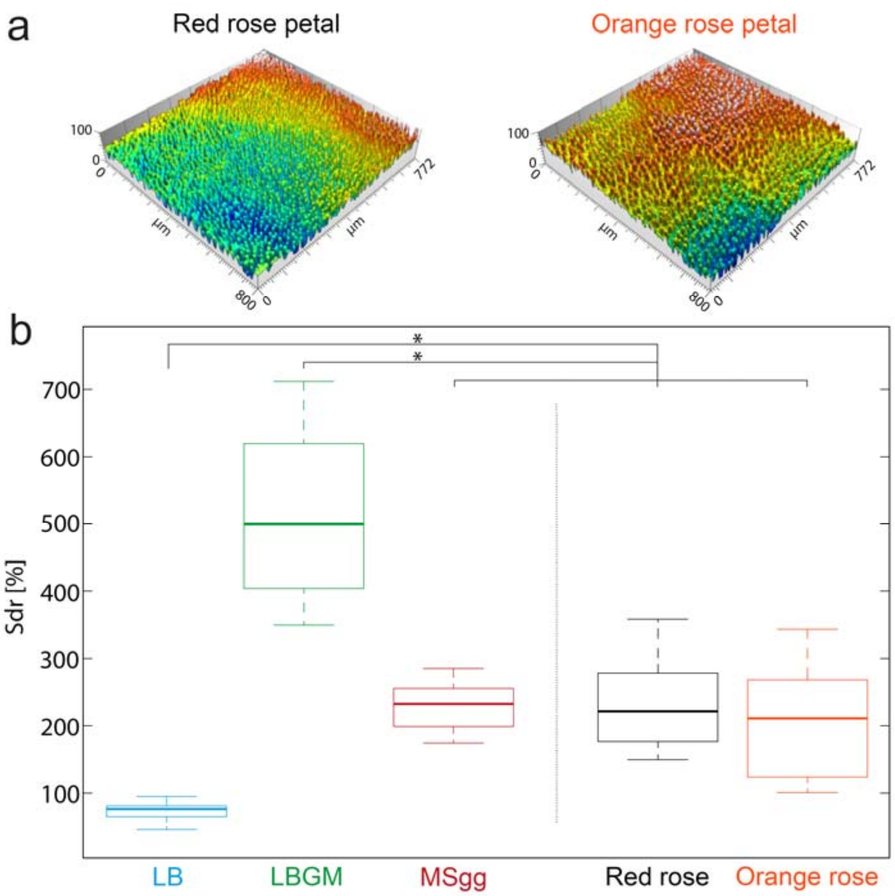

Extended Data Fig. 9 | The surface topology of rose petals is similar to that of *B. subtilis* NCIB 3610 biofilms grown on MSgg agar. **a**, Also for rose petals, images of the surface topology were obtained with light profilometry. A soft correction method was applied when needed, i.e., to remove isolated outliers, outliers around the edges, and measurement noise. The removed outliers were replaced by a smooth shape calculated from the neighbors, resulting in a much accurate surface image. **b**, Similar to MSgg biofilms, the petals of red and orange roses show hydrophobic properties with high droplet adhesion. The box plots contain data from 14 images acquired from a minimum of 4 rose petals. Asterisks denote statistical significance ($p < 0.05$).

References

65 Ueda, K., Seki, T., Kudo, T., Yoshida, T. & Kataoka, M. Two distinct mechanisms cause heterogeneity of 16S rRNA. *J. Bacteriol.* **181**, 78-82 (1999).

66 Verhamme, D. T., Murray, E. J. & Stanley-Wall, N. R. DegU and Spo0A jointly control transcription of two loci required for complex colony development by *Bacillus subtilis*. *J. Bacteriol.* **191**, 100-108 (2009).

67 Hobley, L. *et al.* BslA is a self-assembling bacterial hydrophobin that coats the *Bacillus subtilis* biofilm. *PNAS* **110**, 13600-13605 (2013).

68 Takamatsu, H. *et al.* A spore coat protein, CotS, of *Bacillus subtilis* is synthesized under the regulation of ς(K) and GerE during development and is located in the inner coat layer of spores. *J. Bacteriol.* **180**, 2968-2974 (1998).

69 Shida, T., Hattori, H., Ise, F. & Sekiguchi, J. Overexpression, purification, and characterization of *Bacillus subtilis* N-Acetylmuramoyl-L-alanine amidase CwlC. *Biosci., Biotechnol., and Biochem.* **64**, 1522-1525 (2000).

70 Smith, R. L., Thompson, L. J. & Maguire, M. E. Cloning and characterization of MgtE, a putative new class of Mg2+ transporter from *Bacillus firmus* OF4. *J. Bacteriol.* **177**, 1233-1238 (1995).
